# A Treatise on the Physical Cause of the Death of Christ, and Its Relation to the Principles and Practice of Christianity

**Published:** 1848-01

**Authors:** 


### REVIEWS.

ART. IV.—*A Treatise on the Physical Cause of the Death of Christ, and its relation to the Principles and Practice of Christianity.* By WILLIAM STROUD, M.D. London. 1847.

Great absurdities have grown out of the attempt to mingle philosophy and religion; and yet a greater error could not be committed than to suppose that they are irreconcilable. They are necessarily harmonious, when properly interpreted and understood. It is only a "heretical religion" and a "fabulous philosophy," that make the "unwholesome mixture" which Lord Bacon condemns. Truth is a unit, and can never be self-contradictory. All that is true in science must be in harmony with the teachings of revelation, and in time will come to be so regarded, however for a season the two may be held up as in opposition. It was indeed an unhappy union, when, in the time of Galileo, the defenders of the faith, as they esteemed themselves, attempted to make Astronomy conform to their conceptions of religious truth; but no one reverences his Bible less, because these erring men were overthrown, and their interpretation of its meaning shown to be absurd. Revelation, which seemed to be assailed by astronomy, has found in that sublime science one of its allies and supports. And, from the nature of the case, so it must be with all physical science. Whatever may be the apparent tendency of any new truth in astronomy or geology, while still immature or only partially understood, in time, as it comes to be fully developed, it is sure to be found in accordance with divine truth.

One of the offices of science, and one of its most pleasing offices, is to illustrate and confirm those great principles which have their full elucidation in the sacred scriptures. The author of the learned work before us, with this conception of what is due to sacred things from the medical profession, has undertaken to show how physiology sanctions and sustains the story of the Cross. The subject, if we are not mistaken, is one which will awaken an interest in the mind of every reader. It is treated by Dr. Stroud with candor, ability, and judgment, and if his theory of the phenomena of that great event should fail to satisfy every one, no one can rise from a perusal of his pages without deeper convictions of the reality of the event in all its momentous particulars.

The chief object of the work is stated by the author himself in the following words:

"To demonstrate an important physical fact connected with the death of Christ, and to point out its relation to the principles and practice of Christianity; but, although the subjects discussed and the conclusions deduced from them are, it is hoped, of no inconsiderable value in a devotional point of view, the treatise itself is rather argumentative than sentimental, and more concerned with the foundation of evangelical religion than with its superstructure. The fact is not indeed now announced for the first time, having been more or less correctly anticipated by several pious and excellent writers during the last century; but, as in matters of such solemn import conjecture and probability are not a sufficient ground for conviction, the author has labored to supply a demonstration of the fact, which he trusts will be found both new and satisfactory. He has accordingly been careful not to assume any thing which is not generally acknowledged; and has supported every point of the argument with proofs and evidences so combined, as apparently to leave no other alternative than that which is here maintained. Should the attempt have been successful, it will furnish a fresh proof of the value of inductive reasoning; which, like a sounding line let down into the ocean of time, has thus, from the depth of eighteen hundred years, brought up to the surface a pearl of great price."

The question, it will be seen, is physiological, and therefore one which the studies of medical men especially fit them to elucidate. Intense mental emotions are well known to exert a powerful, and sometimes a fatal influence upon the heart and bloodvessels. Instances are cited by Dr. Stroud from various authors, some of which we give:

"It is observed by Baron Haller, the father of modern physiology, that excessive grief occasions palpitation, and sometimes sudden death; that the corporeal effects of anger and terror are nearly alike, including increased strength, and violent motions both in the heart and throughout the body, and producing *bloody sweats*, and other kinds of hæmorrhage.—'Anger,' says Senac, 'has in certain cases torn the fibres of the heart, and even opened the ventricles. It is not therefore extraordinary that it should be followed by palpitation, and accordingly, various physicians have observed such a result. * * * But fear and terror are not less powerful causes, especially when they seize suddenly. In that case the nerves act with violence on the heart, and derange the order of its movements. The blood is at the same time propelled in these passions by a general shock, or commotion of all parts of the body: it therefore necessarily accumulates in the two trunks of the venæ cavæ, rushes into the auricles, and overcharges them, as well as the ventricles. Here then are two causes, one the consequence of the other, which, as is proved by numerous examples, produced palpitation. Dilatations are, as we have already stated, frequent results of fits of passion. Grief and sadness do not act so suddenly, nor with equal force; but as we have said, these secret and silent passions induce similar disorder.'—'If any one,' remarks Corvisart, 'can seriously deny, or even doubt the fatal physical influence of the passions on the heart, let it suffice him to know that a fit of anger, may produce rupture of the heart, and cause sudden death. * * * Complete rupture of the heart has rarely been observed in the sound state of this organ: some examples may however be cited of this lesion, in consequence of a violent effort, a fit of anger, an epileptic paroxysm, &c. * * * But of all the causes capable of producing organic diseases in general, and more especially those of the heart, the most powerful beyond dispute are mental affections. * * * No mental affection can indeed be experienced without the movement of the heart being either augmented, accelerated, retarded, weakened, or disturbed, without its force in fact being increased, enfeebled, or almost annihilated. Pleasure, pain, fear, anger, in short all the powerful passions, cause the heart to palpitate, to beat more or less frequently, strongly, slowly, or regularly, or to suspend its action momentarily, sometimes even mortally."

The *bloody sweat*, recorded by the Evangelists as one of the effects of the extreme mental anguish of the Redeemer, is a rare effect of moral emotion, but, at the same time, one perfectly well authenticated. Our author adduces the following instances, in the way of illustration:

"The eminent French historian De Thou mentions the case of 'an Italian officer who commanded at Monte-Maro, a fortress of Piedmont, during the warfare in 1552, between Henry II. of France and the Emperor Charles V. This officer having been treacherously seized by order of the hostile general, and threatened with public execution unless he surrendered the place, was so agitated at the prospect of an ignominious death, that he sweated blood from every part of his body.'—The same writer relates a similar occurrence in the person of a young Florentine at Rome, unjustly put to death by order of Pope Sixtus V. in the beginning of his reign, and concludes the narrative as follows: 'When the youth was led forth to execution, he excited the commiseration of many, and through excess of grief, was observed to shed bloody tears, and to discharge blood instead of sweat from his whole body; a circumstance which many regarded as a certain proof that nature condemned the severity of a sentence so cruelly hastened, and invoked vengeance against the magistrate himself, as therein guilty of murder.' Amongst several other examples given in the Ephemerides, of bloody tears and bloody sweat occasioned by extreme fear, more especially the fear of death, may be mentioned that of 'a young boy who, having taken part in a crime for which two of his elder brothers were hanged, was exposed to public view under the gallows on which they were executed, and was thereupon observed to sweat blood from his whole body.'—In his Commentaries on the four Gospels, Maldonato refers to 'a robust and healthy man at Paris who, on hearing the sentence of death passed on him, was covered with a bloody sweat.'—Zacchias mentions a young man who was similarly affected on being condemned to the flames. Schenck cites from a martyrology the case of 'a nun who fell into the hands of soldiers; and, on seeing herself encompassed with swords and daggers threatening instant death, was so terrified and agitated, that she discharged blood from every part of her body, and died of hæmorrhage in the sight of her assailants;'—and Tissot reports from a respectable journal that of 'a sailor who was so alarmed by a storm, that through fear he fell down, and his face sweated blood, which during the whole continuance of the storm returned like ordinary sweat, as fast as it was wiped away.'"

Haller in several passages alludes to the exudation of a bloody fluid from the cutaneous vessels. One of the most remarkable instances of this sort occurred in the person of Charles IX. of France. It is recorded by his historian, "that blood gushed from all the outlets of his body, *even from the pores of his skin;* so that, on one occasion, he was found bathed in a bloody sweat." Dr. Stroud adds to these the following case:

"A young woman, aged twenty-one years, irregular in menstruation, and of indolent habits, and obstinate temper, had been much irritated by some reflections made by her parents, on account of her abjuring the Protestant religion. She left her parental roof, and after wandering about for some time, took up her residence in an hospital. She was then suffering violent attacks of hysteria, attended with general convulsions, and exquisite sensibility in the pubic and hypogastric regions. After paroxysms of hysteria, which sometimes lasted twenty-four or thirty-six hours, this female fell into a kind of ecstacy, in which she lay with her eyes fixed, sensibility and motion suspended. Sometimes she muttered a prayer, but the most remarkable phenomenon was an exudation of blood from the cheeks and the epigastrium in the form of perspiration. The blood exuded in drops, and tinged the linen. The cutaneous surface appeared injected in those parts whence the blood escaped, being red, and showing a network of arborescent vessels. This bloody perspiration took place whenever the hysteric paroxysm lasted a considerable time. This state continued for three months, and ultimately gave way, it is said, to local bleeding, * * * together with strong revulsive measures."

Having established this phenomenon as one of the occasional effects of intense passion, whether of rage or of grief, the author proceeds to demonstrate the influence of such emotions upon the heart. A mortal lesion of this organ, and even actual rupture, he proves, may occur from this cause. To find authorities for it he is not under the necessity of going farther back than our own times, as the following extracts show:

"'It is generally in the left ventricle,' remarks Dr. Hope, 'that the rupture [of the heart] takes place; a circumstance which at first appears remarkable, since this ventricle is the stronger, but for the same reason it contracts more energetically, and * * * it is only strong muscles which undergo rupture from the energy of their own contraction. Hence rupture of the auricles is much more rare than that of the ventricles. The exciting causes of rupture are generally considerable efforts, paroxysms of passion, external violence, as falls, &c. * * * Rupture of the heart or great vessels into the pericardium is not always immediately fatal, as a solid coagulum or a fibrinous concretion has in several instances been known to arrest the hemorrhage for a few hours. Of ten cases mentioned by Bayle, eight died instantaneously, one in about two hours, and another in fourteen. Amongst the cases of rupture of the heart, Dr. Copland enumerates—'violent mental emotions, especially anger, fright, terror, unexpected disappointment, distressing intelligence abruptly communicated, anxiety, &c., sudden and violent muscular efforts, and laborious or prolonged physical exertions of any kind, particularly in constrained positions. * * * In some cases,' he observes,—'inexpressible anxiety and pain have been felt in the præcordia and epigastrium, with cold extremities, and cramps, shortly before dissolution. In the majority rupture has produced instant death, but in some this has not been the case. * * * In most of the cases in which the rupture is preceded by violent pain, M. Oliver thinks that it is produced gradually from the successive laceration of several layers or fasciculi of muscular fibres, and that the pericardium becomes only gradually distended by the effused blood. Where the laceration and aperture are at once large, a copious effusion instantly occurs, fills the pericardium, and abolishes the contractions of the organs."

Of the effect of strong passion upon the heart it would be easy to cite numerous instances. The following is reported by Dr. Townsend, of New York,—the case of a young female who died suddenly in consequence of anguish of mind:

"On dissection, the sac of the pericardium was found filled with about ten ounces of coagulated blood, and two of serum. The heart, on all sides covered by it, was of the ordinary volume, but much loaded with fat. At the summit of the aortic [left] ventricle was discovered the breach, from which the effused blood had issued. It was irregularly lacerated, and measured about half an inch in diameter."

Dr. Stroud relates the particulars of a somewhat similar case, communicated to him by Dr. Williams, of Southampton:

"R. W., a laborer, aged fifty-six years, had generally enjoyed good health, but for ten years had suffered great despondency of mind, owing to the unfaithfulness of his wife. About six months before his death he was troubled with severe cough, which came on in paroxysms, generally at night and early in the morning, and after a fit of this kind was found one morning dead. A post-mortem examination took place in the presence of Mr. Boulton, surgeon of Leamington. On opening the chest, the bag of the pericardium appeared much distended with fluid, and was of a dark blue color. On cutting into it, a pint at least of transparent serum issued out, leaving the crassamentum firmly attached to the anterior surface of the heart. On further examination to ascertain the source of this hæmorrhage, we found the left ventricle, from the origin of the aorta downwards to within an inch of the apex, ruptured. The heart appeared in no way disorganized, there was no softness of its walls, the internal membrane was healthy, and so were the valves of each cavity."

Ludwig, in his *Adversaria*, records a case of the rupture of the right auricle of the heart from violence, in which the following appearances were observed:

"The pericardium was so distended by a large quantity of transparent serum and coagulated blood as to push the lungs upwards. The yellowish serum contained in its cavity exceeded half a pound."

Besides the cases now quoted, Dr. Stroud refers to many others, in all which the extravasated fluid is stated to have been distinctly separated into its crassamentum and serum,—*blood and water.* The bearing of these facts upon the question before us will be easily recognised. Before taking leave of this part of the subject, we make another quotation to show that a quantity of bloody fluid has been found within the pericardium. even when there was no discoverable rupture of the heart or large blood-vessels. Two cases of the kind are related in the German Ephemerides.

"Both the subjects were robust soldiers who died of excessive joy, in whose bodies no morbid condition was afterwards found, except a large quantity of clotted blood in the pericardium, by which the action of the heart had been suppressed. The latter author ascribes the effect to sudden distension of the exhalants opening on the inner surface of the membrane This would correspond to the manner in which bloody sweat is produced; but, as the exhalants of the pericardium are very inferior both in size and activity to those of the skin, it is more probable that in such cases the effusion is due either to rupture of some of the nutrient vessels of the heart itself, termed its coronary vessels, or to hæmorrhage from without, penetrating by a minute or circuitous passage into its capsule. Such at least is the opinion of Morgagni, Zecchinelli, and other anatomists. Of blood thus finding its way into the pericardium by a small aperture, which, without great attention, might easily escape notice, the former gives several examples; and in the Ephemerides, Dr. Daniel Fischer mentions the case of a soldier who died suddenly after eating a hearty dinner, and in whose body the only morbid appearance discovered on inspection was, 'the pericardium filled and distended with very fluid and florid blood. The membrane having been divided longitudinally, in order to trace more exactly the source of the hæmorrhage, this was found at the base of the heart, where a branch of the coronary artery had ruptured, and from which blood was still actually flowing.'"

A word on the mode of capital punishment resorted to in the case of Jesus. Crucifixion was practised among many nations from the remotest antiquity. The chief baker of Pharaoh, according to Josephus, was crucified, and this is probably the earliest instance of crucifixion on record. This mode of execution was not in practice among the Jews, but was instituted by their conquerors, the Romans. Christ was condemned by a Roman tribunal, and was put to death by Roman law. By the Romans crucifixion was regarded as the most ignominious and disgraceful of all punishments, and was rarely practised by them except upon their slaves for capital offences.. "Every thing was thus combined to render the death of the Savior accursed in the sight of the people. He suffered as the vilest of malefactors according to the law of the heathen masters of Judea, for alleged *sedition* against the authority of Cæsar; while, at the same time, his being 'nailed to the tree' represented and fulfilled, in the eyes of the Jews, the divine malediction that was always associated in their minds with the peculiar punishment for *blasphemy*—the pretext, it will be remembered, on which they sought His life."

Death by this mode was protracted and lingering, as might be gathered from the construction of the cross, of which the following description is given:

"The Cross consisted of a strong upright post, sharpened at the lower end by which it was fixed in the ground, having a short bar or stake projecting from its middle, and a long transverse beam firmly joined near its top. As the middle bar, although an important appendage, has been almost universally overlooked by modern authors, it will be proper here to insert the account given of it by some of the early fathers of the church, and founded on personal observation. 'The structure of the cross,' says Irenæus, 'has five ends or summits, two in length, two in breadth, and one in the middle, on which the crucified person rests.' Justin Martyr, in like manner speaks of 'that end projecting from the middle [of the upright post] like a horn, on which crucified persons are seated;' and the language of Tertullian, who wrote a little later, exactly corresponds: 'A part, and indeed a principal part, of the cross is any post which is fixed in an upright position; but to us the entire cross is imputed, including its transverse beam, and the projecting bar which serves as a seat.' The criminal condemned to this dreadful mode of death, having first been scourged, was compelled to carry the cross on his shoulders to the place of execution, a circumstance which implies that the scourging was not excessively severe, and that the dimensions of the gibbet did not in general much exceed those of the human body. On arriving at the spot, he was stripped of his clothes, and after receiving a cup of wine, sometimes medicated with a view to impart firmness or alleviate pain, was speedily nailed to the cross, either before or after its erection. In either case he was made to sit astride on the middle bar; and his limbs having been extended and bound with cords, were finally secured by large iron spikes driven through their extremities, the hands to the transverse beam, and the feet to the upright post."

Dr. Stroud remarks as follows upon the degree and usual duration of the sufferings inflicted by this dreadful punishment:

"The bodily sufferings attending this punishment were doubtless great, but, either through ignorance or design, have been much exaggerated. The insertion of the cross into its hole or socket, when the criminal was previously attached to it, did not necessarily produce the violent concussion which has been supposed; and as the body rested on a bar, it did not bear with its own weight on the perforated extremities. At all events, there have been many examples of persons enduring these sufferings with the utmost fortitude, and almost without a complaint, until relieved from them by death. A fact of importance to be known, but which has not been sufficiently regarded, is that crucifixion was a very lingering punishment, and proved fatal not so much by loss of blood, since the wounds in the hands and feet did not lacerate any large vessel, and were nearly closed by the nails which produced them, as by the slow process of nervous irritation and exhaustion. This would of course be liable to variety, depending on differences of age, sex, constitution, and other circumstances; but for persons to live two or more days on the cross was a common occurrence, and there are even instances of some who, having been taken down in time and carefully treated, recovered and survived. In many cases death was partly induced by hunger and thirst, the vicissitudes of heat and cold, or the attacks of ravenous birds and beasts; and in others was designedly accelerated by burning, stoning, suffocation, breaking the bones, or piercing the vital organs."

Accordiug to Origen and others, the sufferers usually survived about two days. Modern travelers relate instances of crucifixion, of which the following are interesting:

"'The capital punishments inflicted in Soudan,' observes Captain Clapperton, writing in 1824, 'are beheading, impaling and crucifixion; the first being reserved for Mahometans, and the other two practised on Pagans. I was told, as a matter of curiosity, that wretches on the cross generally linger three days before death puts an end to their sufferings.'—When describing the punishments used in Madagascar, the Rev. Mr. Ellis remarks: 'In a few cases of great enormity a sort of crucifixion has been resorted to; and in addition to this, burning or roasting at a slow fire, kept at some distance from the sufferer, has completed the horrors of this miserable death. * * * In the year 1825, a man was condemned to crucifixion who had murdered a female for the sake of stealing her child. He carried the child for sale to the public market, where the infant was recognised, and the murderer detected. He bore his punishment in the most hardened manner, avenging himself by all the violence he was capable of exercising upon those who dragged him to the place of execution. Not a single groan escaped him during the period he was nailed to the wood, nor while the cross was fixed upright in the earth. The wooden frame used in the place of a cross resembles a gallows. To this the malefactor is nailed whilst it remains flat upon the earth, after which it is lifted up with its miserable burden, and fixed in two holes made in the ground for the purpose. Here the sufferer is kept until he dies of cold, hunger, or agony. Some criminals, after being nailed to the frame, have remained for hours for the gaze of the multitude. A fire has oftentimes been placed to the windward of them, by which hey and the cross have been consumed together.'"

Another traveller gives the following account of the execution of the captain of banditti, at Constantinople, a few years ago:

"As a preparatory exercise, he was suspended by his arms for twelve hours. * * * The following day a hook was thrust into his side, by which he was suspended to a tree, and there hung enduring the agony of thirst till the third evening, when death closed the scene; but before that about an hour the birds, already considering him their own, had alighted on his brow to peck his eyes. During this frightful period he uttered no unmanly complaints, only repeated several times—'Had I known that I was to suffer this infernal death, I would never have done what I have. From the moment I led the klephte's life, I had death before my eyes, and was prepared to meet it, but I expected to die as my predecessors, by decapitation.'"

From these statements it is reasonable to infer that the death of our Savior, within six hours after he was nailed to the "accursed tree," was not the result of extreme bodily torture, and therefore, as remarked by our author, that, "although the ordinary sufferings of crucifixion contributed to his death, they were not its immediate cause." It is manifest from the inspired narrative, that the bystanders were surprised at the suddenness of the event, and that Pilate could scarcely believe it had occurrred, when Joseph applied to him for the body of Jesus. Admitting, then, that there was something unusual and extraordinary in the quickness with which the sufferings of our Lord came to a close, can any plausible explanation be offered to account for it?

Two have been proposed:—one, that the Redeemer, seeing his work accomplished, by an act of his own divine will, yielded up his life;—the other, that some mortal lesion of a vital organ suddenly supervened, and was the physical cause of his death. The first has been adopted by the majority of commentators, and is the one generally received; but Dr. Stroud, while admitting the sovereign power of the divine Sufferer to give up or to retain his life, embraces the second. He has these observations on the subject:

"That it was in the power of Christ to avoid such a death, had he chosen to renounce the object of his mission, is evident amongst other reasons from his miraculous overthrow of the hostile band in the garden of Gethsemane; from his question to Peter,—'Thinkest thou that I cannot even now request my Father, and he would send to my aid more than twelve legions of angels? [but] how then would the Scriptures be fulfilled, [which declare] that thus it must be?'—and from his remark to Pilate,—'Thou wouldst not have had any authority at all against me, had it not been given thee from above.'—In all scriptural allusions to the subject, the death intimated, although voluntary, is moreover represented, not as self-inflicted, but as penal and vicarious. In the very passage which has thus been misinterpreted, the death encountered by the good shepherd for the safety of his flock is ascribed to the wolf from whom the hireling flees."

The narrative of what transpired in the garden of Gethsemane, when, in the agony of our blessed Savior, "his sweat was as it were great drops of blood falling down to the ground," is familiar to the reader. Our author thus comments upon the incidents of that awful hour:

"The intense grief and consternation which the Saviour experienced at the commencement of his sufferings in the garden, and under the shock of which he fell prostrate to the earth, might possibly have destroyed him by simple exhaustion, but would never have produced the bloody sweat reported by St. Luke; who, independently of his guidance by the Holy Spirit, was, as a physician, peculiarly well qualified to notice and record such an occurrence. He therefore ascribes this sweat to a cause by which it is fully and solely explained, namely, the communication of supernatural strength;—'There appeared to him an angel from heaven, strengthening him.'—It was then that—'falling into an agony, [Christ] prayed most earnestly, and his sweat became as it were clots of blood dropping to the ground;' implying that he was no longer prostrate as at first, but on his knees. Attempts have been made to explain away the strong terms used by the evangelist, but they certainly denote a sweat mixed with blood in a half-coagulated state, so profuse as to fall from the head and neck (the parts liable to be uncovered, and from which sweat of any kind is most readily furnished,) in thick and heavy drops to the ground. Unless St. Luke meant to convey this meaning, his employment of such expressions is unaccountable. The fact is well stated by M'Lean—[Christ] 'is said to be *in an agony.* An agony is a conflict of nature in the extremity of distress. The Lord was now bruising him, and putting him to grief. So great was the agony and conflict of h s soul, that it produced the most wonderful effect upon his body; for we are told that 'his sweat was as it were great drops of blood falling down to the ground.' A common sweat in the open air, and exposed to the cold damp of night, when those within doors require a fire of coals to warm them, must have been the effect of very great fear and agony. What then must his agony have been, which induced a bloody sweat, and so copious as to fall down in great drops to the ground?'—It was then that, as intimated by the Apostle Paul, 'he offered prayers and supplications, [accompanied] with tears and loud cries, to him who was able to save him from death, and was heard on account of his pious fear;'—in other words, these peculiar and overwhelming sufferings were by divine interposition suddenly terminated, leaving him with restored strength, ready to undergo the trials which next awaited him."

He continues thus:

"On both occasions, these sufferings were distinguished from all others, by beginning and ending abruptly, as well as by their peculiar circumstances and effects. On both occasions, the gloom which oppressed the Redeemer's soul was by divine appointment accompanied by external darkness, as its appropriate sign and illustration. When He was in the garden the preceding evening, it appears from astronomical calculation that the paschal full moon underwent a natural eclipse; on which account, perhaps, the numerous party which went forth to seize him were provided with lanterns and torches. Twelve hours later on the same day, according to the Jewish mode of reckoning, a preternatural darkness overspread the whole land from the sixth to the ninth hour."

Blood and water issued from the side of the Savior when pierced by the spear of the Roman soldier, to account for which many conjectures have at different times been proposed. By the ancient commentators it was generally ascribed to a miraculous interposition. Several modern ones have ascribed it to "serous effusion either in the pericardial or pleural sacs naturally induced by extreme debility," which they suppose to have attended the Saviour's death. This is not the place for discussing the first of these explanations, and there are strong reasons for rejecting the second. The quantity of serum found in the pericardium, in a healthy state, is conceded by anatomists to be small. John and Charles Bell remark:

"If a person have labored under a continued weakness, or have been long diseased, if a person have lain long on his death-bed, if the body have been long kept after death, there is both a condensation of the natural halitus in all parts of the body, and an exudation of thin lymph from every vessel, there is water found in every cavity from the ventricles of the brain to the cavity of the ankle-joint, and so in the pericardium amongst the rest. But, if you open a living animal, as a dog, or if you open suddenly the body of suicides, or if you have brought to the dissecting room the body of a criminal who has just been hanged, there is not in the pericardium one single particle of water to be found."

After some forms of violent death the pericardial fluid is found increased, and is then either pure or tinged with blood. "An effusion of the latter kind is said to have been noticed in stags killed after a hard chase; and in some rare instances of sudden death, occasioned by strong mental emotion, the pericardium has been found distended with blood, owing probably, as Morgagni suspected, to organic disease, and the rupture of vessels; but for the statement of the Grüners that, after death accompanied with anxiety, the pericardium is full of water, there is no evidence.."

But death from this cause would not be sudden, as it was in this memorable instance, but lingering, and preceded by symptoms of oppression and suffocation. The narrative of the sacred text leaves upon the mind the impression, that the discharge of blood and water was considerable, and the distinction between the two so well marked, as to have been perceived by persons at a distance. Dr. Stroud remarks upon this point:

"Bloody serum, whether originally effused in that state, or resulting from subsequent mixture, would not have presented this character; for it would neither have issued rapidly, nor in sufficient quantity, nor would its distinction from ordinary blood have been so striking as to have attracted the attention of an uninformed and somewhat distant spectator. Moreover, unless blood has been previously extravasated, little or none can by any kind of wound be extracted from a dead body, except by the action of gravity, the heart being usually empty, or, if otherwise, devoid of power to expel its contents. This important fact, overlooked by most other writers, was perceived and acknowledged by the Grüners, who nevertheless failed to discover the true explanation, and were induced to adopt the inadmissible opinion, that Christ was not actually dead when pierced by the soldier's spear, but merely in a faint and languid condition, which allowed the heart to act feebly, and, on being wounded, to pour forth its blood, preceded by the water, which they suppose had previously collected in the pericardium."

And this brings us to the conclusion at which our author arrives from the whole inquiry, which is given in the subjoined extract:

"It may, therefore, with certainty be affirmed, that between the agony of mind which the Saviour endured in the garden of Gethsemane, and the profuse sweat mixed with clotted blood which so rapidly followed it, violent palpitation of the heart must necessarily have intervened; this being the only known condition which could have been at once the effect of the former occurrence, and the cause of the latter. In like manner, when on the cross this agony was renewed, and by the addition of bodily suffering, was increased to the utmost intensity, no other known condition could have formed the connecting link between that mental anguish and His sudden death,—preceded by loud exclamations, and followed by an effusion of blood and water from His side when afterwards pierced with a spear—than the aggravation even to ruptur eof the same violent action of the heart, of which the previous palpitation and bloody sweat were but a lower degree and a natural prelude. If, whilst every other explanation hitherto offered has been shown to be untenable, the cause now assigned for the death of Christ—namely, Rupture of the Heart from Agony of Mind—has been proved to be the result of an actual power in nature, fully adequate to the effect, really present without counteraction, minutely agreeing with all the facts of the case, and necessarily implied by them, this cause must, according to the principles of inductive reasoning, be regarded as demonstrated."

Dr. Stroud does not pretend that this explanation is now brought forward for the first time, but on the contrary shows, that, substantially, it has been offered by several commentators, ancient and modern. But want of space forbids any further discussion of the interesting subject.

